# Passive Wireless Pressure Gradient Measurement System for Fluid Flow Analysis

**DOI:** 10.3390/s23052525

**Published:** 2023-02-24

**Authors:** Partha P. Dutta, Alexander C. Benken, Tao Li, John Richard Ordonez-Varela, Yogesh B. Gianchandani

**Affiliations:** 1 Center for Wireless Integrated MicroSensing and Systems (WIMS^2^), ECE Division, EECS Department, University of Michigan, Ann Arbor, MI 48109, USA; 2 Department of Electrical and Computer Engineering, University of Cincinnati, Cincinnati, OH 45219, USA; 3 TotalEnergies, Centre Scientifique et Technique Jean Féger (CSTJF), Av. Larribau, CEDEX, 64018 Pau, France

**Keywords:** physical sensors, energy industry, high resolution, differential, harsh environment

## Abstract

Using distributed MEMS pressure sensors to measure small flow rates in high resistance fluidic channels is fraught with challenges far beyond the performance of the pressure sensing element. In a typical core-flood experiment, which may last several months, flow-induced pressure gradients are generated in porous rock core samples wrapped in a polymer sheath. Measuring these pressure gradients along the flow path requires high resolution pressure measurement while contending with difficult test conditions such as large bias pressures (up to 20 bar) and temperatures (up to 125 °C), as well as the presence of corrosive fluids. This work is directed at a system for using passive wireless inductive-capacitive (LC) pressure sensors that are distributed along the flow path to measure the pressure gradient. The sensors are wirelessly interrogated with readout electronics placed exterior to the polymer sheath for continuous monitoring of experiments. Using microfabricated pressure sensors that are smaller than ø15 × 3.0 mm^3^, an LC sensor design model for minimizing pressure resolution, accounting for sensor packaging and environmental artifacts is investigated and experimentally validated. A test setup, built to provide fluid-flow pressure differentials to LC sensors with conditions that mimic placement of the sensors within the wall of the sheath, is used to test the system. Experimental results show the microsystem operating over full-scale pressure range of 20,700 mbar and temperatures up to 125 °C, while achieving pressure resolution of <1 mbar, and resolving gradients of 10–30 mL/min, which are typical in core-flood experiments.

## 1. Introduction

As the need for wireless, compact, and cost-effective sensing solutions rises in demand [1,2,3,4], microelectromechanical systems (MEMS) become a necessary replacement for conventional macro-scale sensors [5,6]. Considered one of the greatest successes of the MEMS industry, pressure sensors have been widely utilized in industrial, automotive, and healthcare sectors [7,8,9]. Prior work has primarily focused on exploration of pressure sensing methodologies [10,11,12,13] such as piezoresistive [14], capacitive [15], micro-plasma discharge [16,17], and optical fiber Fabry–Perot [18,19], along with specific advancements in sensor properties, such as reduction in size and temperature coefficients or improvements in sensitivity over a large full-scale range [20]. While both piezoresistive and capacitive sensing technologies are widely manufacturable, capacitive pressure sensors allow scalable precision over a wide operating temperature range [21,22]. Capacitive pressure sensors with multiple sensing diaphragms also improve sensitivity and reduce noise [23,24]. Over the years, several research investigations have been directed at fluid flow using microfabricated pressure sensors; these range from directing flow through an orifice in a piezoresistive pressure sensing diaphragm [25] to using a differential pressure sensor to measure pressure drop across two sensing nodes along a flow channel [26]. While these efforts have significantly advanced our capabilities to study pipe flow rates and flow-induced pressure gradients, understanding these gradients across multiple nodes along a long flow channel, with varying fluidic resistance, and at small flow rates remains challenging. Gradient determination requires a full system solution which extracts miniscule pressure differentials across multiple sensing nodes that are precisely located. In addition, environmental constraints such as a large baseline pressure head, a broad operating temperature range, and corrosive test fluids influence the pressure resolution through the overall system design strategy, integration, and packaging, far beyond the sole capabilities of the pressure sensing element.

Whereas a variety of fluidic systems may benefit from the type of distributed pressure gradient measurement capability described above, core-flood experiments present an interesting test case. Core-flood experiments refer to the study of the porosity and permeability of rock core samples by subjecting them to pressurized fluid flow; they are used in the energy industry to plan and assess oil and natural gas recovery strategies [27,28]. The rock core sample is encased in a polymer sheath and then placed inside a high pressure (HP) chamber test cell, with lateral pressure up to 250 bar and temperature up to 125 °C [29]. Fluids are pumped into the rock core longitudinally at pressure levels up to 20 bar. Although the resulting pressure gradients provide valuable information, the measurement locations are limited to the ends of the core. Pressure measurements along the length of the rock core could improve insight as well as permit early detection of failure in these tests, which sometimes extend for months [30]. In past work, X-ray CT scans [31] were used for understanding spatial distribution of fluids and minerals along the rock core during a core-flood experiment. The recorded images helped explain the formation of vugs and wormholes inside the core, thus providing information on the changing permeability of the rock core. Contrary to this high-cost approach which uses custom test setups and image processing algorithms, the physical measurement of forming pressure gradients along the rock core can provide a low cost, continuous monitoring alternate to extract changing rock core permeability information, derived from Darcy’s law [32], as simply represented for a continuous porous medium by Equation (1):

(1) $$K=\frac{\Phi \mu_{f}\Delta L}{A\Delta P}$$

where *K* denotes permeability of rock core, *Φ* denotes flow rate of test fluid, *A* denotes cross-sectional area of rock core, *μ_f_* denotes viscosity of test fluid, and Δ*P* denotes measured pressure gradient across a Δ*L* length of the rock core.

In order to realize a pressure gradient measurement system that addresses core-flood experiments, several challenges must be addressed: Any interference with the fluid flow that is caused by the presence of the sensor must be minimized. This necessitates that only miniature sensing elements be placed along the flow path (at interface between the rock core and polymer sheath), whereas all readout electronics must be placed exterior to the flow chamber. The electrical lead transfer from sensor to interrogation electronics through flow path boundary across the polymer sheath must allow a hermetic seal. The sensing elements must be packaged in a manner that allows pressure to be transmitted through the package, while providing the necessary robustness to withstand large mechanical forces during rock core insertion into polymer sheath, a large baseline pressure head during the core-flood experiment, and the presence of corrosive test fluids such as brine and organic solvents.

In the presence of high lateral pressure and temperature inside the flow chamber, the sensors must measure pressure gradients on the order of few mbar induced by the small flow rates, which are typically 10–30 mL/min. One method for overcoming these problems is to employ an inductive-capacitive (LC) transduction method. This approach utilizes a fully passive sensing element, permitting great reduction in sensor size by eliminating the largest components (e.g., battery and electronics) from the sensed environment [33]. Furthermore, it removes the requirement of a wired connection between sensor and readout electronics [34]. However, its success depends on the integration strategy for interface electronics, which must interrogate the LC sensor to determine the pressure-dependent resonant peak [35], and perform the necessary signal conditioning and post-processing, and a robust sensor packaging approach which preserves the necessary pressure sensitivity and resolution.

**Figure 1:**
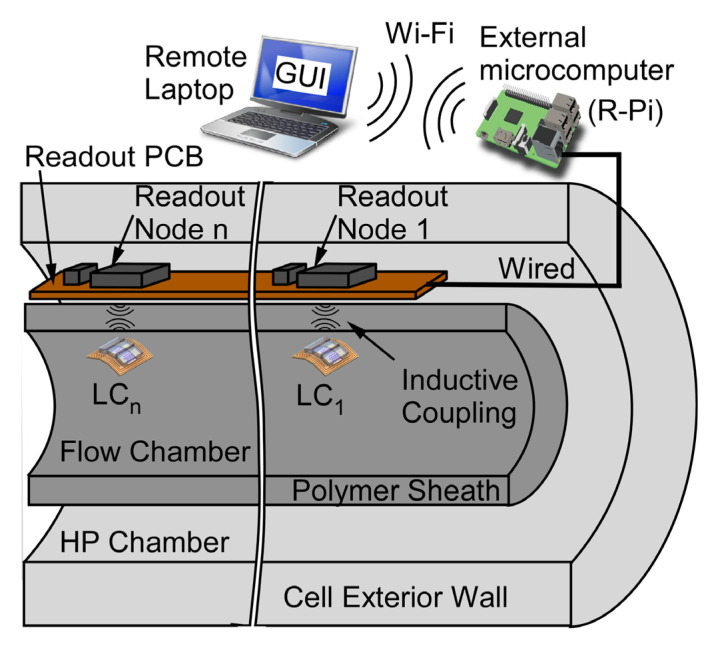
Cross-sectional view of a passive wireless pressure gradient measurement (PGM) system. LC sensors and readout nodes can be scaled to *n* elements, *n* = 4 in this work.

This paper describes a passive wireless pressure gradient measurement (PGM) system, shown in Figure 1, that uses wireless LC pressure sensing elements (denoted as LC sensors, LC_n_), each comprised of a capacitive pressure transducer and planar inductive coil, and corresponding readout nodes to remotely interrogate individual LC sensors. Each readout node consists of an inductor coil, standing wave ratio bridge circuit, and sinusoidal excitation circuitry; a microcontroller unit (MCU) controls each node and digitizes the collected data. An external Raspberry Pi^TM^ (R-Pi) microcomputer (Raspberry Pi^TM^ Foundation, UK) interfaces with the MCU, collecting stored data and running processing algorithms to extract pressure dependent resonant frequency. A laptop connected to R-Pi permits user control through graphical user interface (GUI) and can upload data to the cloud for further processing and remote retrieval.

Although this approach does not limit the number of LC sensors that can be incorporated within a PGM system, the manifestation described in this paper uses four LC sensors incorporated into a flow test setup, wirelessly interrogated by a readout printed circuit board (PCB), and an external control unit located outside the flow chamber. The PGM system was successfully used to measure flow-induced pressure gradients with a pressure resolution of <1 mbar, measuring ≈ 10 mbar pressure drop between adjacent sensors at a flow rate of 14 mL/min representing values for a typical core-flood experiment. If deployed in a core-flood setup, the four LC sensors would be arranged along the rock core within the polymer sheath in the flow chamber, readout PCB with interrogation electronics may be placed on the outside of the polymer sheath within the HP chamber, and external control unit may be placed outside the test cell to permit real-time user interface. The system design is described in Section 2, while fabrication and packaging are described in Section 3. Experimental test results are described in Section 4, followed by discussion in Section 5, with a conclusion and summary in Section 6.

## 2. System Design

This section describes the main components and design methodology used in realizing the PGM system. The first subsection provides the circuit model and mathematical background of the inductor-capacitor (LC) sensor and the readout circuit. Based on this mathematical model and analysis, the LC sensor design strategy is used in the second subsection to obtain a figure of merit that is related to the pressure resolution of the LC sensor; this figure of merit accounts for impact of LC sensor packaging materials and deployment environment. The final subsection addresses the readout circuit design as realized on a flexible printed circuit board.

### 2.1. LC Sensor Model

**Figure 2:**
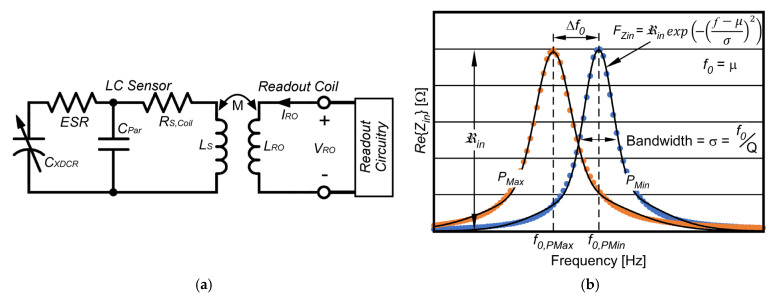
(**a**) Wireless inductive Pressure Gradient Measurement (PGM) System circuit model illustrating the LC sensor and readout coil and circuitry. (**b**) Two *Re*{*Z_in_*} datasets illustrating relevant parameters.

The pressure sensing element of the PGM system is the LC sensor, comprised of a capacitive pressure transducer, *P_XDCR_*, with variable capacitance, *C_XDCR_*, and inductive coil with inductance, *L_S_*; a simplified circuit model is shown in Figure 2a. Here, *C_Par_* is parasitic capacitance of an LC sensor; *R_S_*_,*Coil*_ is parasitic series resistance of an LC sensor coil. The equivalent series resistance, *ESR*, results from resistance of thin film metal electrodes of *P_XDCR_*. Relevant design equations are summarized in Table 1.

The resonant frequency of the LC sensor, *f*_0_ (2) is sensitive to pressure through the changes in sensor capacitance, *C_XDCR_*. When the readout coil with inductance, *L_RO_*, and LC sensor coil with inductance, *L_S_*, are in close proximity, they become coupled, resulting in mutual inductance, *M* (4). When the readout coil is excited with input voltage, *V_RO_* and input current, *I_RO_*, with a resulting complex input impedance, *Z_in_* (3), the pressure-dependent resonant frequency, *f*_0_, of the mutually coupled LC sensor, manifests as a peak in the real part of the input impedance, *Re*{*Z_in_*}, of the readout coil. The value of *Re*{*Z_in_*} is monitored by the readout circuitry, described below.

To extract the pressure dependent *f*_0_ of the LC sensor, a set of discrete data points are collected, where each data point is the magnitude of *Re*{*Z_in_*} at the specific interrogated frequency. Two typical captured datasets of *Re*{*Z_in_*} are shown in Figure 2b; a Gaussian curve, referred to as fitted frequency response waveform, *F_Zin_* (8) is computed for each dataset. From this, *f*_0_ can be determined through interpolation. This method allows frequency resolution to be achieved without requiring collection of an intractably large number of data points.

Bandwidth, σ, is defined as *f*_0_/*Q*, and mean (*µ*) is defined as *f*_0_. In this work, σ is defined as the full width at half maximum, in the frequency span of the *Re*{*Z_in_*} dataset. ℜ_in_, defined in Equation (9), is determined by first identifying the maximum value of *Re*{*Z_in_*}, found at approximately *f*_0_, and minimum value of *Re*{*Z_in_*}, found far away from *f*_0_ (at approximately *f*_0_/10); it can also be estimated using *M*, *Q*, *L_RO_*, and *L_S_*. The quality factor, *Q*, defined in (10), is *f*_0_ divided by σ; it can also be estimated using total effective resistance, *R_S_* (6), *L_S_*, and total capacitance, *C_S_* (7). The shift in resonant frequency, Δ*f*_0_, with applied pressure, Δ*P*, (caused by the change in capacitance, Δ*C*, of the transducer over the full-scale pressure range, Δ*P_FS_*) is defined as Absolute Response, *AR*, (11). AR when normalized to the bandwidth, σ is defined as Relative Response, *RR*, (12).

### 2.2. Design Methodology

In an initial effort to minimize the resolvable pressure of the PGM system, a balance must be found between ℜ_in_, *Q*, and *AR*; furthermore, both the readout coil and the LC sensor must be designed in tandem in order to ensure *M* is large enough such that ℜ_in_ is above the minimum detectable signal for the utilized readout electronics. In designing the LC sensor, there are two main factors that must be considered: (1) *f*_0_ is extracted through curve fitting of discrete data points; and (2) ℜ_in_, *Q* and *AR* are often in direct conflict—as one is increased, the other is reduced. (For example, *Q* can be increased by adding parallel capacitance, *additional C_Par_*, to reduce *R_S_* contributed by transducer *ESR*, but this addition will result in a reduction in *AR*). This inverse relationship between *AR* and *Q* balances *RR* which depends on the product of these terms. The final design parameters must remain within the physical boundary conditions of the specific application; the boundary conditions applicable to the core-flood experiments targeted in this work are shown in Table 2.

The frequency range over which *Re*{*Z_in_*} is measured from an LC sensor (i.e., the interrogation frequency range, *IFR*) must be wide enough so that curve fitting to extract *f*_0_ can be completed. To capture >99% of the sensor readout across the full-scale pressure, a frequency range of approximately ±3σ plus the span of Δ*f*_0_ is required. In addition to *IFR*, the step size between data points (frequency step size, *f_ss_*) must be chosen. With fewer data points, data collection bandwidth can be improved but curve fitting performance (and resolution) may decrease. Empirical testing with the implemented curve fitting algorithm within the R-Pi microcomputer revealed that performance saturated beyond a data density of approximately 10 points within the LC sensor frequency response bandwidth, σ (*f*_0_/Q). Therefore, a *f_ss_* of σ/10 (*f*_0_/(10 *Q*)) is used.

**Figure 3:**
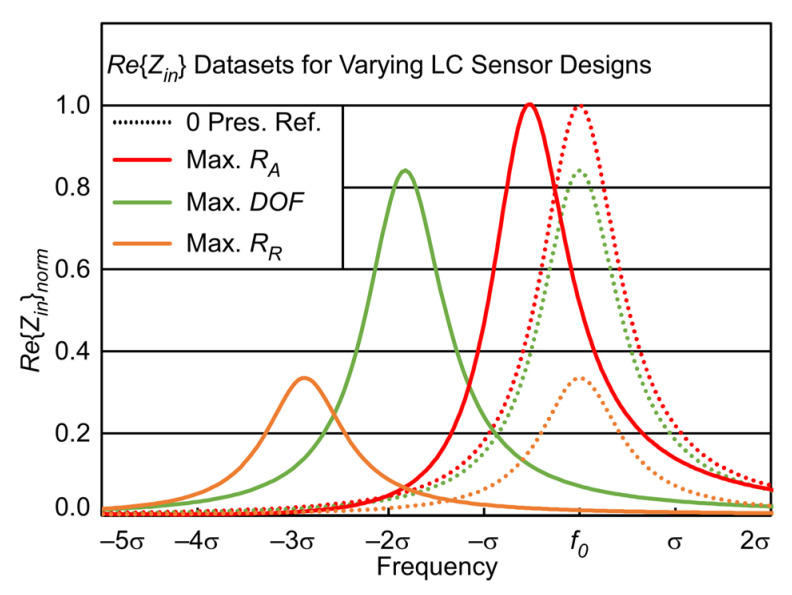
Comparison of example *Re*{*Z_in_*} datasets at 0 and 20 bar applied pressure for three LC sensor designs (given in Table 3). All datasets plotted over their bandwidth (i.e., bandwidth normalized).

The bandwidth normalization in *RR* facilitates the comparison of different LC sensor *AR*, to select the most suitable design. With the discrete nature of the captured dataset of *Re*{*Z_in_*}, it is important to note that, regardless of absolute values of *Q* and σ, the *Re*{*Z_in_*} datasets for all LC sensors look identical over their *IFR*, when bandwidth normalization is utilized. Therefore, the frequency shift due to applied pressure for a given LC sensor design may be then easily expressed in terms of their respective signal bandwidth, as shown in Figure 3.

As previously discussed, in addition to *AR* and *Q* (now captured in *RR*), ℜ_in_ also impacts resolution. Assuming a constant white noise across the frequency spectrum, ℜ_in_ is directly proportional to SNR. In other words, if ℜ_in_ is low, such as due to low mutual coupling between sensor and readout coil (low *M*), the ability of the readout circuit to accurately decipher *f*_0_ through curve fitting shall be low. Thus, ℜ_in_ must exceed a minimum threshold governed by the capabilities of the readout circuit and curve fitting algorithm to allow accurate *f*_0_ capture, thus being proportional to SNR. Increasing ℜ_in_ can be accomplished by increasing *M* and/or *Q* of LC sensor. *M* is primarily dependent on physical parameters (such as turns and diameter) and spacing and orientation between sensor and readout inductors. Whereas *Q* can be increased by increasing *L_S_* or decreasing *C_S_*, it is primarily impacted by reducing *R_S_*. ℜ_in_ and minimum resolvable pressure were approximately determined to bear an empirically linear relationship (i.e., a 2× increase in ℜ_in_ will improve resolution by ≈2×).

To further illustrate how *Q*, *RR*, *AR*, and ℜ_in_ are interconnected, Figure 3 shows a SPICE simulation of three *Re*{*Z_in_*} datasets for different designs using parameters as listed in Table 3. *Re*{*Z_in_*} is plotted over each designs normalized bandwidth, σ. Figure 3 shows the shift in *Re*{*Z_in_*} for an applied pressure of 20 bar; the designs individually maximize Absolute Response, *AR* (red), and Relative Response, *RR* (orange).

The design maximizing *AR* has the smallest *RR* (as its σ is >14× larger than the other designs). The design maximizing *RR* results in the smallest ℜ_in_ (due to high *R_S_* of its *L_S_* coil). However, the design maximizing *FOM*, which is the product of *RR* and ℜ_in,_ Equation (13), results in middling *RR* and ℜ_in_ values, shown in green. This parameter is suitable as a figure of merit because it accounts for the combined impact of *Q* and *AR* through *RR*, Equation (12), as well as the impact of ℜ_in_ on the SNR. The design with the maximum value returned by the *FOM* is the LC sensor design which will theoretically permit the minimum resolvable pressure. The inverse of *FOM* is proportional to pressure resolution; when a fitted proportionality factor, *C*, is included, absolute values for predicted pressure resolution, *Resol_Sim_*, can be found, Equation (14).

An LC sensor design optimization program was implemented in MATLAB to sweep physical LC sensor and readout coil design parameters within their boundary conditions (Table 2) and automatically calculate the *FOM* for each design. Approximations for inductance [36] and coil resistance [37] were utilized while *k* and *M* were determined through finite element analysis (FEA) modeling in the absence of accurate closed form equations.

**Figure 4:**
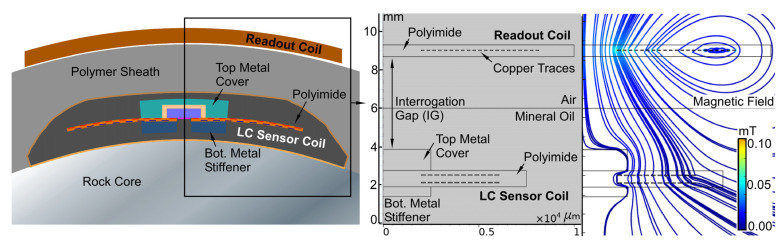
Model geometry of LC sensor and readout coils and Magnetic field distribution at 13 MHz excitation. Assumed material properties for FEA given in Table 4.

The simplified closed form equations that may be used to estimate *M* do not account for the magnetic coupling and leakage flux [38]; additionally, the magnetic behavior of the packaging materials and other nonidealities have an impact on the value. Consequently, FEA was utilized in COMSOL Multiphysics^®^ to extract *M* between *L_S_* and *L_RO_*. Figure 4 shows the 2D axisymmetric model geometry, including the package structure; materials of the LC sensor are noted detailed in Section 3 and listed in Table 4. The magnetic field lines between readout coil and LC sensor at an excitation frequency of 13 MHz are also shown in Figure 4. The field lines show the impact of the top metal cover and bottom metal stiffener: the bending of field lines in the presence of these elements reduces coupling between the LC sensor and center of the readout coil, moving the maximum field line concentration to the outer edge of the LC sensor coil.

In the context of this work, measured pressure resolution, *Resol_Meas_*, is defined as the RMS error (e.g., ±standard deviation) of at least 50 readings (*n* ≥ 50) of the LC sensor taken by the PGM system. The measured pressure resolution, *Resol_Meas_* was calculated by the RMS error of *f*_0_ by *AR* converting to units of pressure, (15):

(15) $$\;Resol_{Meas}=\frac{RMS\;Error\;of\;f_{0}}{AR}$$

**Figure 5:**
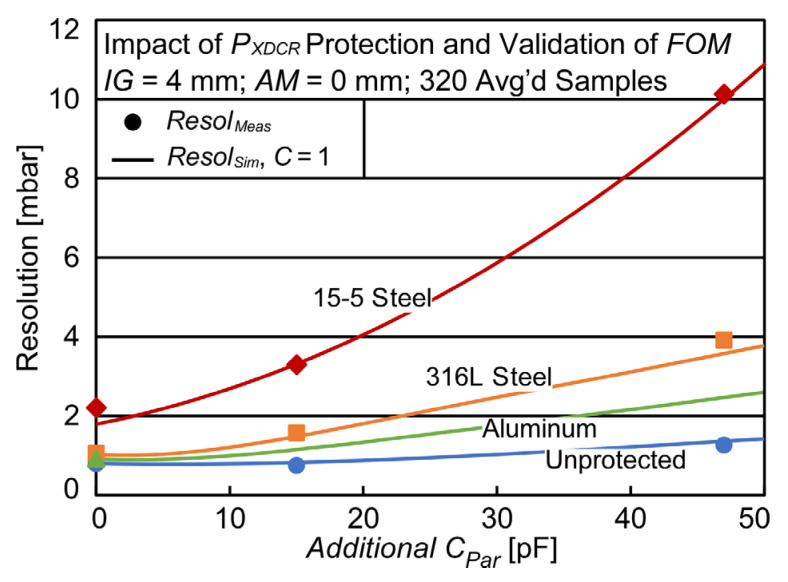
Verification of *FOM* comparing measured and predicted pressure resolution for varying LC sensor designs.

In order to verify FOM, four types of LC sensors were fabricated on planar inductor coils using varying *P_XDCR_* protection materials, and ceramic capacitors were electrically connected in parallel with *C_XDCR_* to emulate varying *C_Par_* values, resulting in ten distinct designs. A total of 50 discrete datasets for all ten designs were taken and used to both calculate *Resol_Meas_* and fit *C_Par_* and *R_S,Coil_* values to the LC sensor circuit in Figure 2a (using previously measured values for *C_XDCR_*, *ESR*, *L_S_*, and *L_RO_*) via SPICE modeling. These component values were coded into the MATLAB model and the additional *C_Par_* value was swept from 0 to 50 pF while returning *Resol_Sim_* (*C* = 1). The *Resol_Sim_* and *Resol_Meas_* for these designs are plotted in Figure 5, showing an agreement within 11%, confirming the ability of the proposed *FOM* to reliably predict pressure resolution. Whereas the resolution of the unprotected LC sensor varied from ≈1 mbar (with 0 pF additional *C_Par_*) to ≈1.5 mbar (with 50 pF additional *C_Par_*), the resolution of an LC sensor protected with 15–5 stainless steel had a larger variation from ≈2 mbar (with 0 pF additional *C_Par_*) to ≈12 mbar (with 50 pF additional *C_Par_*).

The study yielded the preferred LC sensor design—corresponding to the highest value of *FOM*, which was 0.515—a double layer inductor with a coil outer diameter of 13 mm and 17 turns, with a trace width and spacing of 0.125 mm. The associated readout inductor design had an outer coil diameter of 17.5 mm and 21 turns, with trace width and spacing of 0.15 mm.

### 2.3. Readout Circuit

**Figure 6:**
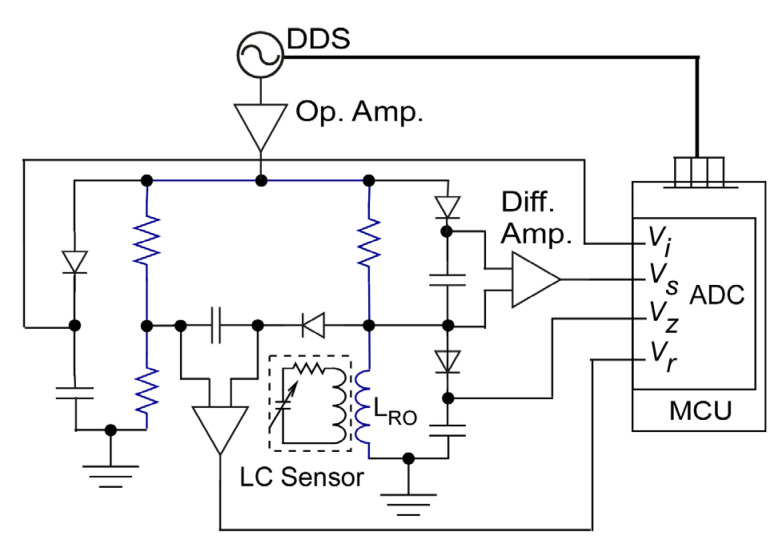
Circuit schematic of readout node on Readout PCB.

As noted earlier and illustrated in Figure 1, individual LC sensors (LC_n_) were wirelessly coupled to specific readout inductor coils integrated into each corresponding readout circuit node (Readout Node n). Each Readout Node, illustrated in Figure 6, was controlled by an individual MCU (C8051F990, Silicon Laboratories, Inc., Austin, TX, USA) with an embedded 12-bit analog to digital converter (ADC). A direct digital synthesizer (DDS) (AD9850, Analog Devices, Inc., Wilmington, MA, USA) generated a sinusoidal signal to excite a standing wave ratio bridge circuit. The readout coil, with its impedance varying because of the coupled LC sensor, formed one branch of the standing wave ratio bridge circuit; the remaining branches were implemented with fixed value resistors. Four voltages of each branch of the standing wave ratio bridge circuit, *V_z_*, *V_s_*, *V_r_*, and *V_i_*, were amplified by differential amplifiers, realized with ADA4891 (Analog Devices, Inc., Wilmington, MA, USA). Amplified signals were digitized using ADCs embedded within the MCU. The *Re*{*Z_in_*} was calculated within the MCU using (16) and (17), where |*V_z_*|, |*V_s_*|, |*V_r_*|, and |*V_i_*| represented peak values of the voltages [39].

(16) Re{Zin}=(|50(|VZ→||Vs→|)|2+502) SWR50 (SWR2+1)

(17) $$SWR=\left(\left|\frac{1}{2}\vec{V_{i}}\right|+\left|\vec{V_{r}}\right|\right)/\left(\left|\frac{1}{2}\vec{V_{i}}\right|-\left|\vec{V_{r}}\right|\right)$$

## 3. System Fabrication and Packaging

### 3.1. LC Sensor Fabrication

**Figure 7:**
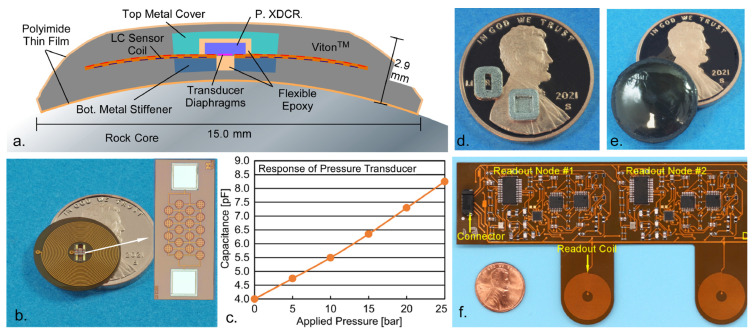
(**a**) Cross-section of packaged LC sensor. (**b**). Unpackaged LC sensor coil and inset image of capacitive pressure transducer. (**c**) Response of capacitive pressure transducer. (**d**). 3D printed bottom metal substrate stiffener (left) and top metal cover (right). (**e**) Fully packaged LC sensor. (**f**) Readout PCB showing two readout nodes.

The LC sensor inductor coil was fabricated on a flexible polyimide printed circuit board (PCB) substrate, manufactured by FlexPCB (Santa Ana, CA, USA). It contained electrical contact pads at the center for mounting the capacitive pressure transducer. The *C_XDCR_* was electrically connected using Duralco 120, a high temperature silver epoxy from Cotronics (Brooklyn, NY, USA). Capacitive pressure transducers used in this work were developed at the University of Michigan [23]. These capacitive pressure transducers provide special properties not found in commercially available transducers such as high sensitivity, low value baseline and parasitic capacitance, low temperature coefficients, and small volume. The pressure range is extended through electrically insulated capacitive electrodes, which can operate in contact mode. Furthermore, the substrate under the diaphragm offers natural over-pressure protection. Figure 7b shows the assembled flexible PCB inductor coil with one capacitive pressure transducer; an inset image of the pressure transducer element is also shown. The response of the transducer due to applied pressure is shown in Figure 7c. The transducer has very low baseline capacitance of ≈4 pF, along with a ≈4.5 pF linear full-scale capacitive response over a 0–25 bar applied pressure range.

### 3.2. LC Sensor Packaging

The LC sensor packaging must be sufficiently robust to provide protection against mechanical forces and withstand environments inside core-flood experiment equipment (containing corrosive chemicals and temperatures up to 125 °C) while still permitting pressure transmission to the transducer. To limit any disturbance in the fluid flow during a core-flood experiment, the packaged sensor may be integrated within the thickness of the polymer sheath encasing the rock core. For a typical polymer sheath thickness of 5 mm, the packaged sensor should be less than 3 mm in thickness to enable integration without compromising the integrity of the polymer sheath. The package is composed of a top metal cover and bottom metal substrate stiffener, Viton^TM^ rubber encapsulation, and thin film polyimide coating; a cross section is shown in Figure 7a.

The top metal cover and bottom metal substrate stiffener (Figure 7d) were used to protect the capacitive pressure transducer, providing mechanical support to prevent any shear forces or static high pressure from causing delamination. They were fabricated by Fathom Advanced Manufacturing (Oakland, CA, USA) using the direct metal laser sintering (DMLS) 3D printing process. An AlSi_10_Mg aluminum alloy [40] was used to both minimize size and reduce impact on the LC sensor coupling to the readout coil while still providing maximum support and protection. The top of the top metal cover and bottom of the bottom metal substrate stiffener were curved to match the curvature radius of the rock core. To secure them to the LC sensor, a high temperature flexible epoxy (Duralco 4538, Brooklyn, NY, USA) was employed. This flexible epoxy also surrounded the transducer and transducer diaphragms, providing additional protection while still permitting pressure to be transmitted to the diaphragms.

**Figure 8:**
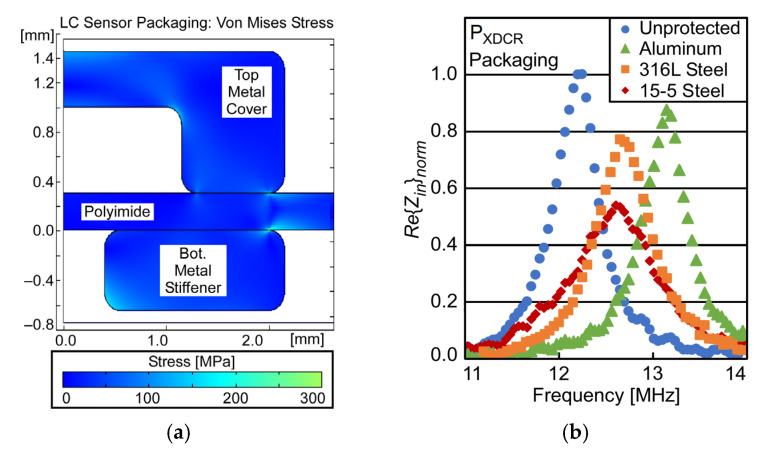
(**a**) FEA modelling of von Mises stress distribution in LC sensor packaging under applied pressure of 230 bar. (**b**) Experimental *Re*{*Z_in_*} datasets for LC sensors in unpackaged and packaged configurations. Assumed material properties for FEA given in Table 4.

To determine the necessary thicknesses of the top metal cover and bottom metal substrate stiffener, an FEA model was developed in COMSOL Multiphysics^®^. This model was used to evaluate the stress distribution and deflection of the AlSi_10_Mg aluminum alloy under high pressure. The simulations showed that the maximum von Mises stress in a 1.2 mm thick AlSi_10_Mg aluminum alloy top metal cover was 90 MPa at 230 bar applied pressure (Figure 8a), permitting a safety factor of >3× for the 300 MPa yield strength of the AlSi_10_Mg aluminum alloy. The maximum deflection of the top metal cover was ≈3.6 μm at 230 bar applied pressure, indicating that the designed clearance of 200 µm was sufficient.

To confirm the strength of the metal cover, a sample of the AlSi_10_Mg top metal cover and bottom metal stiffener were mounted to the *L_S_* coil using Duralco 4538; the dummy LC sensor was pressurized to 250 bar and heated to 125 °C for 120 min. At the conclusion of this test, no visible deformation of the top metal cover and bottom metal stiffener was observed.

For this work, it is important that the top metal cover and bottom metal substrate stiffener are fabricated from materials with relative magnetic permeability (*μ_r_*) as low as possible. Materials with high *μ_r_* placed near the inductor will act as a ferrite core; at high frequencies, this will greatly increase the effective value of *R_S,Coil_*, reducing *Q* and ℜ_in_, worsening pressure resolution. For comparison two additional sets of results are presented here: (i) the results of an LC sensor without top metal cover and bottom metal substrate stiffener installed (unprotected), and (ii) an LC sensor protected with top metal covers and bottom metal substrate stiffeners fabricated from 15-5 stainless steel (*μ_r_* ≈ 95), and 316 L stainless steel (*μ_r_* ≈ 1.4). These are compared to the results from the metal cover of AlSi_10_Mg aluminum alloy (*μ_r_* ≈ 1.0) are shown in Figure 8b. The 15-5 stainless steel results in a *Q* and ℜ_in_ reduction of >50% compared to an unprotected LC sensor; however, the AlSi_10_Mg aluminum alloy results in a reduction of <10%. These reductions in both *Q* and ℜ_in_ combine to reduce pressure resolution significantly. Given that pressure resolution is indirectly proportional to both *Q* and ℜ_in_, (14), a reduction in these values by >50% worsens pressure resolution by >4×; however, a reduction of <10% worsens resolution by <1.2×.

After the top metal cover and bottom metal substrate stiffener were secured, the LC sensors were encapsulated in Viton^TM^ rubber, which is known to tolerate elevated temperature and pressure [41]. This was performed by using a custom mold and Fluorodyn Viton^TM^ Caulk from Thermodyn Global Sealing (Houston, TX, USA). As Viton^TM^ is incompatible with certain solvents used in core-flood experiments, such as toluene, a 100 µm thin film polyimide coating (PI 2610, HD MicroSystems, Wilmington, DE, USA) was applied to the Viton^TM^ surface. A fully packaged LC sensor is shown in Figure 7e.

### 3.3. Readout Circuit Fabrication

The readout PCB, containing four readout coils and associated readout nodes, was fabricated on a flexible polyimide PCB substrate manufactured by FlexPCB (Santa Ana, CA, USA). The readout PCB also included a 5-pin connector for power supply and communication with the external microcomputer. A segment of the fabricated PCB showing the connector and two readout nodes is shown in Figure 7f.

## 4. Test Results

A number of experimental tests were performed to assess the wireless readout and flow gradient measurement capability of the implemented PGM system. A test setup was constructed to enable in-house PGM introducing controlled flow-induced pressure differentials to LC sensors; PGM results were recorded from when the system was integrated into the setup. Operation of the PGM system over the full-scale temperature (25–125 °C) and pressure (0 bar–20 bar) range was verified. Key factors that determine system performance were investigated; these include pressure and temperature calibration of LC sensors, digital improvements through oversampling, and accounting for variation in system integration for use in real world applications.

### 4.1. LC Sensor Readout System

As described in Section 2, LC sensors were interrogated with a coupled readout coil and associated readout node. Ideally, each LC sensor is axially aligned to a readout coil with an *IG* of 4 mm. On receiving a command from the user through the graphical user interface (GUI), the R-Pi external microcomputer serially communicates over an I^2^C bus with the MCU in each readout node to trigger a measurement cycle. During a measurement cycle, the MCU commands the DDS to supply a sinusoidal excitation signal to the standing wave ratio bridge circuit between the *IFR* of 11–15 MHz with an *f_ss_* of 35 kHz. The dwell time at each discrete frequency step is 100 ms, during which the ADC samples the four voltages of the standing wave ratio bridge circuit, collecting and averaging 250 samples to reduce uncorrelated white noise. These four voltages are then used to determine *Re*{*Z_in_*} at each discrete interrogation frequency. After completion of each measurement cycle, *Re*{*Z_in_*} values stored in the MCU Flash memory are transferred to the R-Pi, where a Python-based program extracts *f*_0_ by applying a Gaussian fit, *F_Zin_*, to the *Re*{*Z_in_*} dataset. The total time required by the PGM system to trigger four readout nodes into a measurement cycle, interrogate each LC sensor over the *IFR*, transfer the *Re*{*Z_in_*} dataset from the MCU at each node to the R-Pi, and extract *f*_0_ by fitting *F_Zin_*, was 25 s. The ability of the PGM readout system to accurately extract LC sensor *f*_0_ was characterized using twelve LC sensor loads with *f*_0_ values between 3 and 12 MHz as measured using a benchtop network analyzer (Keysight E5061B). These loads were constructed from planar inductor coils fabricated on standard PCB manufacturing methods and surface mounted ceramic capacitors. The *f*_0_ values of these loads as measured by the PGM readout system were within 6% of the benchtop network analyzer measurements.

### 4.2. Dynamic Pressure Response and Flow Resolution

**Figure 9:**
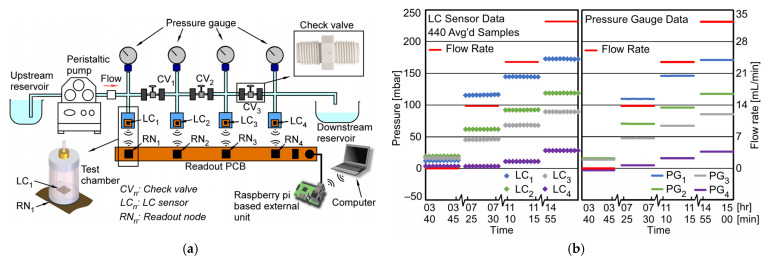
(**a**) Flow test setup illustrating LC sensors (LC_n_) and wired pressure gauges (PG) in flow path with wirelessly coupled Readout nodes (RN_n_). Check valves (CV_n_) create pressure drop. (**b**) Measured pressure of LC sensors (LC_n_) and flow rate during flow experiment at 25 °C (left), and measured pressure of wired gauges (PG_n_) during flow experiment (right). Error bars for measurement resolution not visible at scale (95% confidence interval: <1.5 mbar).

To apply a pressure gradient across multiple LC sensors through fluid flow and record PGM, a custom test setup was constructed (Figure 9a). The setup consisted of a peristaltic pump to generate constant fluid (mineral oil) flow, individual pressure chambers to house LC sensors, LC_n_, (*IG* = 4 mm, *AM* = 0 mm), and check valves, CV_n_, to create known pressure differential, Δ*P_CV_*, between each chamber; commercial pressure gauges, PG_n_, [42] were used to monitor pressure in each LC sensor chamber. Each PG_n_ acts as a high-resolution pressure reference at each sensing node with a limited operating pressure range of 500 mbar. The analog output of each wired PG_n_ was digitized using a commercial data acquisition board (USB-6363-OEM DAQ, National Instruments Corp., USA) controlled in LabVIEW™ (National Instruments Corp., Austin, TX, USA). All fluidic components in the test setup were interconnected using fluidic connectors and polyurethane tubing with an 1/8” I.D. and 3/16” O.D., manufactured by ATP Pneumatics (Milford Center, OH, USA).

During a typical pressure drop calibration of the flow test setup, when a flow rate of ≈32 mL/min was applied, CV_1_ and CV_3_ provided a Δ*P_CV_* of 60 ± 5 mbar, while CV_2_ provided a Δ*P_CV_* of 35 ± 5 mbar. The pressure drop across each check valve (Δ*P_CV_*) was estimated using Equation (18),

(18) $$\Delta P_{CV}=\left(PG_{1}-PG_{2}\right)-\Delta P_{C}$$

where *PG*_1_ and *PG*_2_ are the commercial pressure gauge readouts at the two ends of the check valve and Δ*P_C_* is the pressure drop in the associated fluidic connectors, estimated to be ≈3 mbar at 25 mL/min fluid infusion rate and ≈1 mbar at 5 mL/min infusion rate. Uncertainty in Δ*P_CV_* arises from resolution of commercial gauges, varying pressure head due to consumption of oil in the upstream reservoir, and pressure gauge placement on the opposite side of the pressure chamber relative to the LC sensor. Furthermore, CV’s have a variation in pressure drop of up to 10% [43].

The pressure drop in the polyurethane tube of the test setup, for white mineral oil flow, was estimated using the simplified Hagen-Poiseuille’s equation [44] shown in Equation (19), where ∆*P_T_* is the pressure drop in the tube, *µ_oil_* is the dynamic viscosity of white mineral oil, *L* is the tube length, *Φ* is the flow rate, and *D* is the tube inner diameter. For a tube length of 2.54 cm, tube inner diameter of 3.175 mm, mineral oil density of 838 Kg/m^3^, dynamic viscosity of 0.0103 N-s/m^2^ and flow rate of 25 mL/min, the calculated pressure drop is 0.437 mbar. Using the same assumptions but substituting in a 5 mL/min flow rate, the pressure drop is 0.0873 mbar. Hence, the pressure gradient between two adjacent LC sensor channels *x* and *y* (Δ*PS_xy_*) shown in Equation (20), is the summation of Δ*P_CV_*, Δ*P_C_*, and ∆*P_T_*.

(19) $$\Delta P_{T}=\frac{128\mu_{oil}L\Phi }{\pi D^{4}}$$

(20) $$\Delta PS_{xy}=\Delta P_{CV}+\Delta P_{C}+\Delta P_{T}$$

In a typical PGM experiment, LC sensors were placed into each pressure chamber and axially aligned (axial misalignment, *AM* = 0 mm) to readout coils on the Readout PCB with an interrogation gap, *IG* of 4 mm; *AM* and *IG* are further discussed in Section 4.5. The test setup was then saturated with mineral oil by initiating fluid flow until steady state flow was established; fluid flow was then ceased and measurements were taken (i.e., pressure readings during a flow rate of 0 mL/min). The fluid flow was restarted and increased in three discrete steps of 14 mL/min, 24 mL/min, and 32 mL/min; the flow rate was held constant at each flow step for 225 min. Figure 9b left plot shows a time-series of pressure measurements from four LC sensors (shown with colored diamond markers); 440 samples were averaged per measurement. Fluid flow rate during experiment is plotted with red line. In the presence of flow, LC_1_, being nearest to the pump, recorded the highest pressure head (inlet pressure); as the flow encounters resistance at each check valve, the pressure head drops sequentially from LC_2_ to LC_3_ to LC_4_. Recorded pressure measurements from the commercial pressure gauges (PG_n_) are shown to the right in Figure 9b. The LC sensor measurements are in close agreement with the PG measurements, differing by <5% on average. This difference in pressure between LC sensor and commercial PG measurements can be accounted for since the LC sensor and corresponding PG are not exactly collocated; fluctuations in ambient pressure in the room and flow resistance provided by the tube and connectors between the PG and LC sensor can then result in minor differences between the two pressure levels at the LC sensor and PG.

Although the demonstrated PGM system uses a passive wireless interrogation approach, low power computing, and compact readout electronics on a flexible PCB substrate, the system can successfully decipher very low PGM (e.g., ≈10 mbar between LC_2_ and LC_3_ at 14 mL/min flow rate). These results demonstrate the ability of the PGM system to measure static pressure with a resolution of up to 1.0 mbar when oversampling and averaging is utilized and detect pressure gradients due to fluid flow in a dynamic fluid flow environment. The following subsections illustrate the methodology used to achieve the high pressure measurement resolution which enables PGM using passive LC sensors.

### 4.3. System Static Pressure and Thermal Response

**Figure 10:**
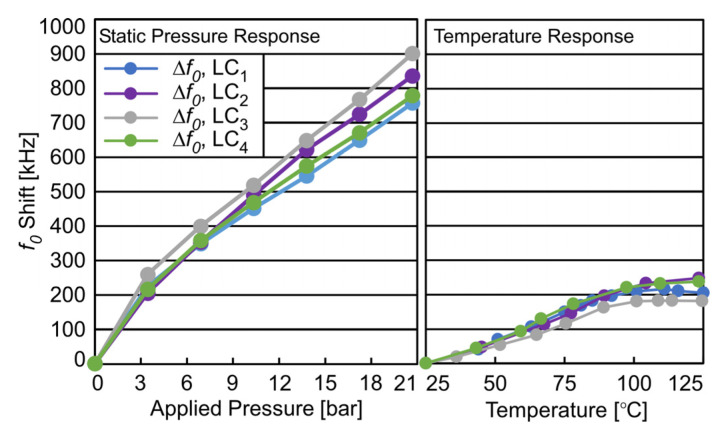
This figure shows the shift in the resonant frequency, *f*_0_, resulting from applied pressure (**left**) and the applied temperature (**right**). Error bars not visible at scale (95% confidence interval: ±0.041 kHz).

To alleviate the impact of variation in parasitic capacitance, planar coil inductance, and capacitance response of each pressure transducer, along with variation originating from LC sensor integration and packaging steps, each packaged LC sensor was individually pressure and temperature calibrated to ensure a high pressure measurement resolution. To perform this calibration operation over the full-scale temperature (25–125 °C) and pressure (0 bar–20 bar) range, LC sensors were axially aligned to each readout coil on the PCB with an *IG* of 4 mm. The PGM system with four LC sensors was placed inside a chamber and pressurized using nitrogen gas. Pressure was raised to 20,700 mbar (300 psi) at 25 °C in steps of 3447 mbar (50 psi). Readings from the four LC sensors were taken at each pressure step and are shown in Figure 10; LC sensors showed an average response of approximately 38 Hz/mbar from 0 bar to 20.7 bar.

The PGM system was then placed into an oven (at atmospheric pressure) and the temperature ramped from 25 °C to 125 °C while readings were continually taken. The system remained fully functional with no change in performance. *f*_0_ shift due to temperature (where a shift of “0” refers to *f*_0_ at 0 applied pressure and ambient temperature of 25 °C) for four LC sensors (LC_1_–LC_4_) over the full-scale temperature range is shown in Figure 10. All LC sensors showed a linear shift in *f*_0_ of ≈2.7 kHz/°C from 25–100 °C, reducing to a shift of approximately 0.0 kHz/°C from 100 °C to 125 °C.

### 4.4. Conversion of Resonant Frequency to Pressure

In order to convert *f*_0_ to applied pressure while also accounting for changes in ambient temperature which also affect *f*_0_, multi-dimensional regression (i.e., higher order polynomial curve fitting) [45] was utilized. This approach involves fitting a 3-D polynomial surface to the LC sensor response, where one dimension is applied pressure, one dimension is ambient temperature, and the third dimension is extracted *f*_0_. A third order multi-dimensional polynomial was fit to the static pressure response as measured over the full-scale pressure range of 0–20.7 bar and temperature range of 25 °C to 125 °C. The calibration equation for conversion of extracted *f*_0_ and temperature, *T*, to applied pressure, *P_App_*, is shown in Equation (21), where *a_1_*–*a_6_* are fitted coefficients using least squares regression to static full-scale pressure and temperature test data.

(21) $$P_{App}=a_{1}{\left(f_{0}\right)}^{3}+a_{2}{\left(f_{0}\right)}^{2}+a_{3}{\left(f_{0}\right)}^{\;}+a_{4}+a_{5}\left(f_{0}\cdot T\right)+a_{6}\left(T\right)$$

The calibration equation was found to convert *f*_0_ and measured temperature with an accuracy of <±0.25% of full-scale (<±50 mbar) over the designed full-scale pressure and temperature range (0–21,000 mbar and 25–125 °C, respectively). However, it is important to note that the accuracy with which the test apparatus could apply pressure to the PGM system was comparable (<±50 mbar or <0.25% of full-scale).

### 4.5. Pressure Resolution Enhancement

**Figure 11:**
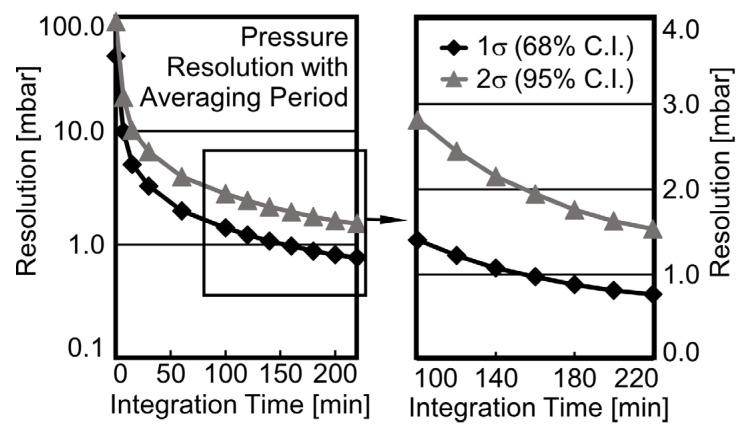
Measured pressure resolution (*Resol_Meas_*) of PGM system.

With the use of additional digital signal processing through oversampling and averaging, in-band noise power can be lowered, thereby improving effective pressure resolution of LC sensors. In the target application, the signal tests can be performed for months, and the signal changes can occur in geological time scales, presenting ample opportunity for this approach. Each quadrupling of the number of averaged samples lowers in-band noise by 6 dB, improving pressure resolution by a factor of two [46]. Figure 11 shows the resulting instantaneous *Resol_Meas_* of 46 mbar permitted by the PGM system along with improvement in pressure resolution when oversampling and averaging were used; when 320 samples were averaged, resolution improved to 1.0 mbar. As seen in the plot, the improvement in pressure resolution with number of averaged samples is rapid to begin with but eventually saturates to a point of diminishing returns; the estimated pressure resolution also being dependent on the chosen confidence interval of measurement. In core-flood experiments, which can be several months in duration, high bandwidth measurements are generally not necessary, permitting a large number of samples to be averaged for resolution improvement without impacting system efficacy.

### 4.6. System Deployment Variations

**Figure 12:**
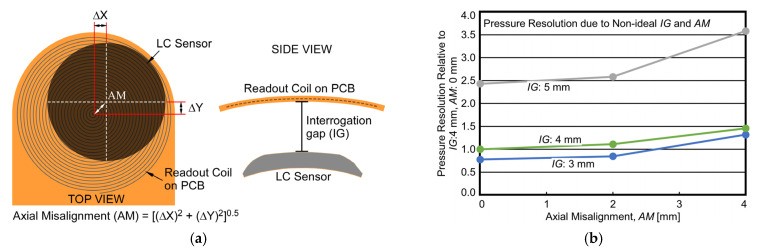
(**a**) Illustration of axial misalignment (*AM*) and interrogation gap (*IG*). (**b**) Measured pressure resolution with non-ideal *AM* and *IG*.

In deploying the PGM system in a core-flood experiment setup, or in other real world PGM applications, it may be more challenging to achieve perfect axial alignment between the LC sensor and its corresponding flexible readout coil. In addition, different applications may necessitate different interrogation gap, *IG* requirement between each LC sensor and readout coil pair. To understand such variations in deployment requirement, the impact of axial misalignment, *AM*, (Figure 12a) of up to 4 mm was also studied as a function of *IG*. In particular, *IG* values of 3 mm, 4 mm, and 5 mm were experimentally evaluated and are plotted in Figure 12b using *AM* of 0 mm and *IG* of 4 mm as a reference. It is important to note that the plot depicts absolute values of pressure resolution relative to the 0 mm *AM*, 4 mm *IG* measurement, i.e., a lower value in the plot translates to a superior pressure resolution. The results show that the presence of both *IG* and *AM* have a significant negative impact on pressure resolution. In the absence of misalignment (with *AM* = 0 mm), as the *IG* was changed 4 mm to 3 mm, the improvement in pressure resolution was ≈25%, and as *IG* was changed 4 mm to 5 mm, the loss in pressure resolution was ≈145%. For a constant interrogation gap (with *IG* = 4 mm), as the *AM* was changed 0 mm to 2 mm, the loss in pressure resolution was ≈10%, and as *IG* was changed 0 mm to 4 mm, the loss in pressure resolution was ≈46%. These results highlight the significance of considering *AM* and *IG* during LC sensor design process to improve performance with changing deployment requirements. For instance, the optimal LC sensor and readout coil design (i.e., the design which maximizes *FOM*) for an *IG* of 4 mm is not necessarily the same for an *IG* of 5 mm. The results also show across various values of *IG*, the loss in pressure resolution for an *AM* up to 2 mm is low (<10%), thus allowing some flexibility during system assembly in a core-flood experiment setup.

## 5. Discussion

In this work, the combined interrogation time for all four nodes was limited by two primary factors: (1) the settling time of the output response, and (2) the available MCU Flash storage (<2 kB) of the digitized |*V_x_*| value data. The settling time was the result of time necessary at each interrogation frequency for the DDS frequency output and standing wave ratio circuit response to be stable before digitization (≈50 ms). This settling time caused a delay at each interrogated frequency; therefore, the total time required to interrogate all 112 frequencies across the full *IFR* was ≈6.5 s (including an additional ≈5 ms to sample the actual |*V_x_*| values). The limited flash memory storage of the MCU required that the digitized |*V_x_*| values be transferred from the MCU to the external microcomputer after each frequency, taking ≈4.5 s. Furthermore, the data transfer between the readout PCB and microcomputer was limited to a single serial communication bus. Because a modular design approach was utilized for the readout circuit (i.e., each readout node contained its own complete circuit), the data transfer speed was further limited, as each of the four individual MCUs was required to wait for the preceding MCU to complete its data transfer to the microcomputer before sending its own data, resulting in a total data transfer period of ≈18.5 s for all four MCUs. In an alternate application scenario requiring higher operational bandwidth (and where the number of readout nodes is limited), further refinement of the standing wave ratio bridge circuit could be performed to reduce settling time; a common excitation and readout electronics platform with a single, higher performance MCU and DDS to control all readout nodes simultaneously may be implemented, which could significantly reduce interrogation time. Use of a single, more computationally capable MCU would enable fitting algorithms to run real-time on the readout PCB itself, significantly reducing the requirement on external processing and data transfer. In terms of the present circuit, an external Flash memory at each readout node could also eliminate the need for repeated communication with the external unit at each frequency step through local data storage, thus reducing overall interrogation time.

The approach used to design the PGM system has notable features that can extend its use to other applications. This work is scalable to a large number of sensors because of the use of a modular readout PCB design. The system hardware and software were co-designed to maximize pressure resolution. For instance, the LC sensor design which maximizes pressure resolution was selected while operating within the capabilities of the deployed curve fitting algorithm. The *FOM* presented in this work encompasses all major design dependencies of the LC sensor and readout system which may be in direct conflict with one another (e.g., interrogation gap, inductor coil diameter, capacitive pressure transducer response, etc.), in context of unique challenges posed by the application (robust packaging, flexible PCB substrate, custom pressure transducers with unique Δ*C/C*_0_ response, misalignment tolerance, interrogation gap, etc.). The same design refinement model for LC sensors can be extended across different PGM applications.

## 6. Conclusions and Summary

This work has focused on the investigation and realization of a passive wireless pressure sensing system using inductive-capacitive (LC) transduction to measure ultra-small flow-induced pressure gradients (≈few mbar) at small flow rates (10–32 mL/min) in high resistance fluidic channels such as a rock core, with a pressure measurement resolution of <1 mbar, while being subjected to a large lateral pressure up to 250 bar and temperature up to 125 °C. The design of the LC sensor and packaging approach, sensor interrogation and readout electronics, data analysis and software enhancement methods, and external interface for user control were presented with application in core-flood experiments. The sensing system demonstrated PGM capability during fluid flow with a ≈10 mbar successful PGM between adjacent LC sensors caused by a 14 mL/min flow rate.

While application driven requirements of a high pressure measurement resolution, which improves PGM and flow analysis, is typically addressed through specific improvements in sensor element and/or interface electronics, this work addressed the same problem through understanding relationships between all system components (including sensor design, sensor packaging, interface electronics, processing capability, firmware, etc.) and their relative impact on overall system performance. The resulting co-design of hardware and software elements lead to a more cost-effective solution. An LC sensor design refinement model for analytically predicting and minimizing pressure resolution, which accounted for both sensor non-idealities and secondary packaging effects, was investigated and experimentally validated. The LC sensor was comprised of a package using 3D printed aluminum caps to protect the capacitive transducer, encased in Viton^TM^ and thin film polyimide to provide chemical resistance to the environment and still permit pressure transduction to the transducer, and measured <ø15 × 3 mm^3^.

The LC sensor interrogation system was comprised of a readout PCB and external unit containing a microcomputer and remote laptop with custom GUI for user control. Interrogation electronics extracted and digitized the real part of the input impedance, *Re*{*Z_in_*}, of an inductor coil inductively coupled to the LC sensor over a set of discrete frequencies. This discrete dataset was then transferred to the external microcomputer which extracted the pressure-dependent resonant frequency, *f*_0_, via interpolation by fitting a continuous Gaussian curve. A custom GUI running on a remotely connected laptop permitted real-time user control of the system. A modular system design approach allows easy customization of number of LC sensors used to fit varying application needs while use of commercial manufacturing practices promote volume scalability of the proposed solution.

The system was characterized over a full-scale pressure range of 0–21,000 mbar and temperature range of 25–125 °C. Measurements of flow-induced pressure gradients were successfully taken by integrating the full system into a custom test setup. A pressure resolution of <1 mbar was demonstrated when oversampling and averaging software enhancement techniques were utilized.

Applications in which measurement of flow-induced pressure gradients were previously thought impossible or impractical are now within the realm of possibility. Performance may be improved by utilizing a higher performance circuitry, such as an MCU with additional memory or an ADC with a higher bit resolution and faster data capture rate. While this work focused on core-flood experiments, the LC sensor design refinement techniques, readout circuit approach, *f*_0_ extraction procedure, and software enhancement methods can easily be used to create passive wireless flow gradient sensing systems to meet the requirements of many different applications such as in high performance liquid chromatography and unmanned navigation of underwater vehicles.

## 7. Patents

Y. Gianchandani, T. Li, P. Dutta, A. Benken, J.-R. Ordonez-Varela, “Distributed Pressure Measurement System for Core Flood Experiments”, disclosure filed 2 June 2019, UM file no. 2019-452, provisional patent 62/910,828 filed 4 October 2019, PCT/US2020/053970 filed 2 October 2020.

## Figures and Tables

**Table 1:** Inductor-Capacitor (LC) Sensor Design Equations.

Parameter	Equation	
Resonant Frequency	$f_{0}=\frac{1}{\sqrt{L_{S}\;\left(C_{XDCR}+C_{Par}\right)}}$	(2)
Complex Input Impedance	$Z_{in}=\frac{V_{RO}}{I_{RO}}$	(3)
Mutual Ind.	$M=k\sqrt{L_{S}\;L_{RO}}$	(4)
Eff. Series Res. Of *C_XDCR_*	$R_{ESR}=\frac{ESR\;{\left(C_{XDCR}\right)}^{2}}{{\left(C_{XDCR}+C_{Par}\right)}^{2}}$	(5)
Total Eff. LC Sensor Res.	$R_{S}=R_{ESR}+R_{S,Coil}$	(6)
Total Eff. LC Sensor Cap.	$C_{S}=C_{XDCR}+C_{Par}$	(7)
Fitted Gaussian Curve	FZin≈Rinexp(−(f−μ)2σ2)=Rinexp(−(f−f0)2(f0Q)2)	(8)
Mag. Of Input Impedance	$R_{in}=Re\left\{Z_{in}\left(f_{0}\right)\right\}-Re\left\{Z_{in}\left(\frac{f_{0}}{10}\right)\right\}\approx \left(\frac{M^{2}Q}{L_{RO}L_{S}}\right)$	(9)
Quality Factor	$Q=\frac{f_{0}}{\sigma }\approx \frac{1}{R_{S}}\sqrt{\frac{L_{S}}{\left(C_{XDCR}+C_{Par}\right)}}=\frac{1}{R_{S}}\sqrt{\frac{L_{S}}{C_{S}}}$	(10)
Absolute Response	$AR=\frac{\Delta f_{0}}{\Delta P_{FS}}\approx \frac{\;{\left(\sqrt{L_{S}\;C_{S}}\right)}^{-1}-{\left(\sqrt{L_{S}\;\left(C_{S}+\Delta C\right)}\right)}^{-1}}{\Delta P_{FS}}$	(11)
Relative Response	$RR=\left(\frac{AR}{\sigma }\right)=\left(\frac{AR\cdot \;Q}{f_{0}}\right)$	(12)
Figure of Merit, *FOM*	$FOM=RR\cdot R_{in}={\left[\frac{AR\cdot \;Q\;\cdot R_{in}}{f_{0}}\right]}_{\;}$	(13)
Simulated Pres. Resol.	$Resol_{Sim}=C\left(\frac{f_{0}}{AR\cdot Q\cdot \;R_{in}}\right)$	(14)

**Table 2:** Design Constraints for LC Sensor and Readout Coil.

Design Parameter	Dimension	Comments
LC sensor, max. diameter	<13 mm	To maintain packaged LC sensor diameter ≤15 mm
*L_RO_*, max. diameter	<25 mm	LC sensor pitch is 50 mm
Min. *L* trace width, spacing	0.125 mm	Min. manufacturable dimension
Nominal Interrogation Gap (*IG*) of *L_S_* and *L_RO_*	4 mm	Polymer sheath thickness in core-flood experiment
Full-scale pressure, Δ*P_FS_*	20 bar	Full-scale pressure range of core-flood experiments
*C_XDCR_* offset cap, *C*_0_	4.0 pF	*C_XDCR_* cap. at 0 app. pres.
*C_XDCR_* cap. change over Δ*P_FS_*, Δ*C_FS_*	3.2 pF	Total capacitance change of *C_XDCR_* over *ΔP_FS_*
*C_XDCR_ ESR*	200 Ω	Equivalent Series Resistance (*ESR*) of *C_XDCR_*
*Interrogation Gap, IG*	4 mm	Vertical spacing between LC sensor and readout coil
*Axial Misalignment, AM*	0–2 mm	*AM* w.r.t. the centers of paired LC sensor and readout coil

**Table 3:** Design Parameters used for Figure 3 Simulations.

	Equ.	Max. *AR* (Red)	Max. *RR* (Orange)	Max. *FOM* (Green)
*L_S_* Diameter	---	ø13.0 mm		
Trace Width	---	0.925 mm	0.125 mm	0.125 mm
Inductor Layers	---	2	4	2
Inductance [μH]	---	0.6	36.4	10.5
*f*_0_ [MHz]	(2)	52.8	6.7	12.5
σ [kHz]	---	7178	167	494
*Q*	(10)	7.3	40.1	25.3
R_in*norm*_	---	1.0	0.35	0.88
*AR* [Hz/mbar]	(11)	0.189	0.024	0.045
*RR* [ppm/mbar]	(12)	827	4163	2662
*FOM_norm_*	(13)	0.33	0.63	1.00

**Table 4:** Assumed material properties for FEA.

Material	Density [kg/m^3^]	Young’s Modulus [GPa]	Poisson’s Ratio	Elect. Cond. [S/m]
Air	-	-	-	1.00 × 10^−2^
Aluminum	2700	70.0	0.33	3.77 × 10^7^
Copper	8700	130	0.34	5.99 × 10^7^
Mineral oil	850	-	-	1.75 × 10^−1^
Polyimide	1300	3.10	0.34	6.66 × 10^−16^
Viton^TM^	1840	0.01	0.47	2.1 × 10^−9^

## Data Availability

The data presented in this study are available on request from the corresponding author. The data are not publicly available.
